# Identifying essential genes in *Schaalia odontolytica* using a saturated transposon library

**DOI:** 10.1128/jb.00164-25

**Published:** 2025-08-01

**Authors:** Joseph K. Bedree, Jacob Bourgeois, Pooja Balani, Lujia Cen, Erik L. Hendrickson, Kristopher A. Kerns, Andrew Camilli, Jeffrey S. McLean, Wenyuan Shi, Xuesong He

**Affiliations:** 1 Section of Oral Biology, Division of Oral Biology and Medicine, School of Dentistry, University of California-Los Angeles 49038, Los Angeles, California, USA; 2 Department of Microbiology, ADA Forsyth Institute, Somerville, Massachusetts, USA; 3 Department of Molecular Biology and Microbiology, Tufts University School of Medicine 12261, Boston, Massachusetts, USA; 4 Department of Oral Medicine, Infection and Immunity, Harvard School of Dental Medicine 124048 , Boston, Massachusetts, USA; 5 Department of Periodontics, School of Dentistry, University of Washington 7284, Seattle, Washington, USA; University of Florida, Gainesville, Florida, USA

**Keywords:** periodontitis, oral microbiome, microbiome, gingivitis, actinomycetes, TM7x, *Saccharibacteria*, Tn-seq, *Nanosynbacter*

## Abstract

The unique epibiotic-parasitic relationship between *Nanosynbacter lyticus* type strain TM7x, a member of the newly identified candidate phyla radiation, now referred to as *Patescibacteria*, and its basibiont, *Schaalia odontolytica* strain XH001 (formerly *Actinomyces odontolyticus*), requires more powerful genetic tools for a deeper understanding of the genetic underpinnings that mediate their obligate relationship. Previous studies have mainly characterized the genomic landscape of XH001 during or post-TM7x infection through comparative genomic or transcriptomic analyses, followed by phenotypic analysis. Comprehensive genetic dissection of the pair is currently cumbersome due to the lack of robust genetic tools in TM7x. However, basic genetic tools are available for XH001, and this study expands the current genetic toolset by developing high-throughput transposon insertion sequencing (Tn-seq). Tn-seq was employed to screen for essential genes in XH001 under laboratory conditions. A highly saturated Tn-seq library was generated with nearly 660,000 unique insertion mutations, averaging one insertion every two–three nucleotides. A total of 203 genes comprising 10.5% of the XH001 genome were identified as putatively essential.

**IMPORTANCE:**

*Schaalia odontolytica* strain XH001, an early colonizer of the oral multispecies biofilm (dental plaque), forms a unique epibiotic-parasitic relationship with *Nanosynbacter lyticus* type strain TM7x, a member of the newly identified *Patescibacteria* (formerly candidate phyla radiation). Achieving a mechanistic understanding of their relationship requires practical genetic tools for dissecting the roles played by different genetic mediators and shedding light on how their interspecies interaction may affect dynamics in the oral microbiome. In this study, we developed a high-throughput mutagenesis technique, Tn-seq, in XH001. The constructed Tn-seq library enabled the identification of putatively essential genes in XH001, revealing growth requirements under laboratory conditions. This library can be leveraged in future studies to elucidate TM7x’s dependence on XH001 at the molecular level.

## INTRODUCTION

Members of the *Patescibacteria* phylum (formerly candidate phyla radiation) live as obligate epibionts of host bacteria, making their isolation and characterization difficult. While several *Saccharibacteria* and host pairs have been recently isolated (1–4), the *Nanosynbacter lyticus* type strain, TM7x (HMT_952), and *Schaalia odontolytica* (formerly *Actinomyces odontolyticus* subsp. *actinosynbacter*) strain XH001 co-culture is the first and most well-studied pair (5–19). Studying their interaction has been stymied due to TM7x’s recalcitrance to growth in independent culture and the paucity of genetic tools for both species. However, transposon and site-specific mutagenesis tools were recently developed for the closely related *Saccharibacteria* spp. (3). Additionally, we developed a site-specific mutagenesis methodology for the basibiont, XH001 (7), which elucidated AI-2 quorum sensing genetic mediators upon TM7x infection that was built upon the work of Yeung et al. who pioneered genetic system development in closely related *Actinomyces* spp. A broad-host range vector (20), pJRD215 (21), and integration vectors were developed for *Actinomyces naeslundii* (22, 23). Marker-less, in-frame deletion mutants were generated for *Actinomyces oris* MG-1 using GalK counterselection (24). Additionally, lytic and temperate bacteriophage were used for transfection in *Actinomyces*; however, none were found to transfect *S. odontolytica* (25). Most recently, newer genetic systems have been built for *A. oris* MG-1 through leveraging marker-less, in-frame mutations using fluorescence (mCherry) counterselection (26) or transposon mutagenesis (27). This cumulative knowledge laid the groundwork for developing a high-throughput mutagenesis tool for the study of XH001, which has been a barrier to identifying essential genes in *Schaalia* spp., even though such studies have been conducted for the major oral pathogens *Aggregibacter actinomycetemcomitans* (28), *Fusobacterium nucleatum* (29, 30), *Porphyromonas gingivalis (31*), and *Streptococcus mutans* (32, 33). Tn-seq (34) is a robust technique for elucidating the essential genes of any bacterium. High-throughput transposon mutagenesis achieves an insertional saturation, allowing a probabilistic assessment of essentiality based on relative gene size and number of insertions (31, 35). The construction of an XH001 Tn-seq library enabled an analysis of essential genes in XH001 and marked a crucial advancement in XH001 genetic tools for the broader scientific community.

## MATERIALS AND METHODS

### Bacterial strains, plasmids, and media

All bacterial strains, plasmids, and media used in this work are listed in Table S1. All growth conditions used followed previous culturing protocols (7), except for XH001::Tn*5* mutants that were selected on brain-heart infusion (BHI) broth or agar (Difco Laboratories, Detroit, Michigan) with 500 µg/mL of kanamycin sulfate (Fisher Bioreagents, Hampton, NH, United States). All strains, whether grown in broth or on agar, were incubated at 37°C in microaerophilic conditions (2% O_2_, 5% CO_2_, balanced with nitrogen) without shaking using Whitely Workstation A35 (Microbiology International, Frederick, Maryland).

### Transposon mutagenesis and library collection

XH001 underwent electrocompetent cell preparation and transposition with the EZ-Tn*5* transposon as previously described (7, 15, 27, 36) with the following modifications: electrocompetent cells underwent a glycine shock (5% v/v final concentration) prior to harvesting, which has been previously shown to increase transformation efficiency (27). Transposon DNA template was generated by amplifying the EZ-Tn*5* transposon with primers ME-9F and ME-9R from the pMOD-2/Kan215 plasmid as previously described (27) and purified via QIAquick PCR Purification Kit (Qiagen) following the manufacturer’s protocol. Next, a transposome master mix reaction containing 2 µL of purified ME- kanR -ME PCR product (~200 ng), 4 µL EZ-Tn5 transposase (Biosearch Technologies), and 2 µL 100% glycerol was incubated for 2 h at room temperature. Subsequently, 200 µL of electrocompetent cells was briefly incubated with 1.5 µL of transposome mixture on ice and transformed, then recovered in 1 mL of BHI at 37°C for 3 h. The entire 1.2 mL culture of recovered transformants was plated 50 µL at a time to optimize cell spread and selected on BHI with 500 µg/mL of kanamycin sulfate for up to 7 days in microaerophilic conditions described above. The resulting colonies from each library were pooled with an inoculation loop as previously described (37) and collected in 10 mL of fresh media supplemented with 500 µg/mL of kanamycin sulfate. Six separate libraries were generated, each comprising three independent transformations to maximize the number of unique EZ-Tn*5* insertions into the chromosome. These six individual libraries were generated in separate transformation experiments, and when collected, they were aliquoted (~2.0 × 10^8 CFUs/mL) and stored in 30% glycerol at −80°C. One cryovial from each library was later used for DNA isolation.

### DNA isolation and deep sequencing

DNA isolation was performed using MasterPure Complete DNA and RNA Purification Kit (Biosearch Technologies) following the manufacturer’s protocol with the following modifications: pre-lysis incubation of samples with Lysozyme (50 mg/mL) and mutanolysin (2 mg/mL) at 37°C and included an RNAase step for 30 min at 37°C. Library preparation was performed as previously described (31, 38) with the following modifications: during the first PCR amplification step, forward primer 1 was added to a reverse primer mixture consisting of primers 2 and 3. This reverse primer reaction mixture consisted of primer 2, a truncated version of primer 3, which was added at a 10:1 volumetric ratio (primer 2:primer 3, respectively) to enhance amplification and avoid primer dimer formation initially observed in PCR reactions with only primers 1 and 3. The second PCR amplification step, the barcode addition, was completed using primer 4 and barcode primers (BC1-12) listed in Table S1. All PCR reactions were completed using Q5 Hot Start High-Fidelity DNA Polymerase (New England Biolabs, Ipswich, MA) following the manufacturer’s protocol. For deep sequencing on Illumina’s HiSeq 2500 platform, a custom sequencing primer was used (Table S1).

### Bioinformatic analysis for gene essentiality

Bioinformatic analysis of the Tn-seq library sequencing data sets was performed as described (35, 39), first comprising trimming off the 3′ poly-C adapter and 5′ Ez-Tn*5* mosaic end (ME) sequences from any given read (sequence) using stringent parameters for cutadapt.py (40). All trimmed reads from each of the six libraries were merged into one file to determine aggregate insertional density using parameters from the previously established approach (35). The aggregate hop count analysis was performed with the following parameters: (i) minimum read positional cutoff of 15 (15 reads at a given position needed for analysis); (ii) when aggregating EZ-Tn*5* hops into the genome, did not count reads in the first or last 5% of the CDS; and (iii) only count reads that align 100% to the reference across the entire read (no mismatches or gaps). For essential gene analysis, the following criteria were used: Dval genome value (defined as the number of reads of each gene divided by the expected number of reads based on gene size) ≤ 0.01 (35) and minimum region length = 100 base pairs, generating a putatively essential gene list (Table S2). All designated CDS gene loci were mapped and assigned to the Clusters of Orthologous Groups (COGs) as previously described (41, 42).

Spearman correlation matrix comparing DvalGenome values between the six individual EZ-Tn*5* mutagenesis libraries assessing all regions within the XH001 genome (intergenic and CDS) was implemented using GraphPad Prism 8 software. Putatively essential genes were visualized using Circos 0.69-9 (https://circos.ca/) (43). All genes were designated by gene name or APY gene loci tags in the light blue outermost ring. Genome insertion snapshots used for highlighting putatively essential genes were taken by displaying transposon insertions along the *S. odontolytica* strain XH001 (LLVT01000001.1) genome in Integrated Genome Viewer (44). Putatively essential protein functional domains were identified for XH001 proteins by searching for the non-uniform distribution of transposon insertions in candidate genes. After identifying the candidate genes, putatively homologous protein functional domains for encoded proteins were identified using InterProScan (https://www.ebi.ac.uk/interpro/result/InterProScan/) (45) and plotted with transposon insertions in Integrated Genome Viewer. COG analysis was performed as previously described (41), and figures were generated using GraphPad Prism 8 software.

The identified putatively essential genes were compared against the Database of Essential Genes (DEG) (46) version 15.2 (http://tubic.tju.edu.cn/deg/) (47) using local BLASTp alignment with an *e*-value threshold of 0.001. The Bacterial and Viral Bioinformatics Resource Center (BV-BRC) has developed a curated list of essential genes using flux-balance analysis (FBA) (48) for a set of reference and representative genomes, which includes *S. odontolytica* ATCC 17982. FBA predicts essential genes by simulating gene knockouts and assessing their impact on a metabolic network. These genes, therefore, are only a computational prediction based on metabolic pathways and are not derived from Tn-seq or other methods. However, in addition to the DEG database comparison, they serve to further validate the essential genes from this study predicted by FBA within the same species as XH001. XH001 proteins were compared to the BV-BRC essential gene predictions for *S. odontolytica* ATCC 17982 using BLASTp alignment with an *e*-value threshold of 0.01.

## RESULTS

### Mutant library construction and essential gene analysis by Tn-seq in XH001

**Fig 1:**
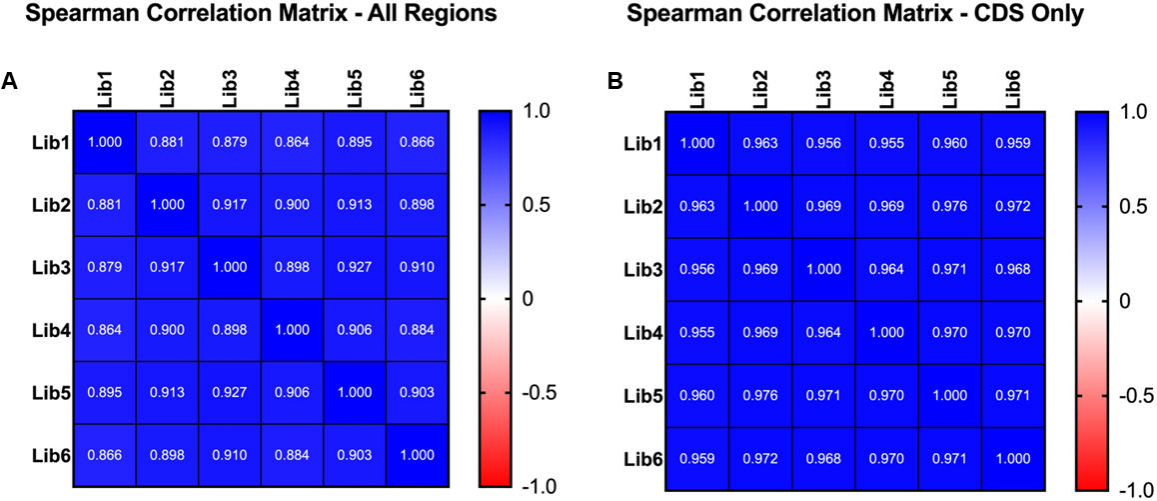
Robust EZ-Tn*5* mutagenesis of the XH001 genome. (**A**) represents the Spearman correlation matrix comparing the DvalGenome values between six individual EZ-Tn*5* mutagenesis experiments assessing all regions within the XH001 genome (intergenic and CDS). (**B**) represents the Spearman correlation matrix comparing the six individual EZ-Tn5 mutagenesis experiments assessing all CDS regions within the XH001 genome. All correlations were significant (>0.8).

To construct a highly saturated Tn-seq library, the EZ-Tn*5* mini-transposon was selected for its ease of use and its stable, random insertions (49, 50) relative to the Himar1-mariner transposon, which has a strong AT-nucleotide insertional bias (34, 51) that is unsuitable for high GC% organisms. While the original Tn*5* normally shows low efficiency (52), a combination of a hyperactive triple mutation of the Tn*5* transposase (49) and inverted repeat ends containing a mosaic end (ME) sequence (53) increased the transposition insertion efficacy by several orders of magnitude (52). Furthermore, it has been used in several bacterial species (27, 35, 54, 55). The kanamycin-resistance cassette from pJRD215 (21) was cloned into the MCS of the commercial EZ-Tn*5* pMOD vector, yielding a custom EZ-Tn*5* transposon, which previously demonstrated high rates of transposition in a closely related *Actinomyces* spp. (27). This amenable transposon system was utilized to generate a highly saturated insertion library in XH001 (Fig. 1). A total of six separate mutant libraries were generated (27) and collected (37) using established protocols with modifications (see Methods). Each mutant library underwent DNA extraction, library preparation, and deep sequencing as previously described (31, 38).

### Analysis of the location and patterns of insertion sites within a highly saturated transposon library reveals insights into the genome

The presence of insertions in a gene, which reflects a mutant bacterium in which transposons were inserted in those positions, indicates the gene is dispensable in the library BHI growth condition. Likewise, the absence of insertions in a gene suggests that the gene is essential in this growth condition. However, in the absence of additional experiments to prove essentiality on a gene-by-gene basis, we refer to these as putatively essential genes in this study. To identify putatively essential genes in the XH001 genome, we aggregated the transposon sequencing data across six biological replicates to eliminate technical variation by following previously described essential gene parameters cutoffs (31). To evaluate our hypothesized, unbiased insertional mutagenesis with the EZ-Tn*5* transposon, we used Spearman’s rank correlation coefficient to compare the DvalGenome values between all six libraries, which revealed consistent insertion saturation results across the replicates (Fig. 1; Table S1). Sequencing showed extensive genome coverage with nearly 660,000 unique insertions, averaging one insertion every two–three nucleotides. We then applied the following subsequent analysis criteria: first, poor quality reads (MAPQ < 1) are not used in the analysis. Next, there must be at least 15 sequenced reads for a position to be counted as an insertion. This was done to reduce false insertion sites, which can occur due to sequencing errors, especially with nearly identical repeated sequences in the genome.

**Fig 2:**
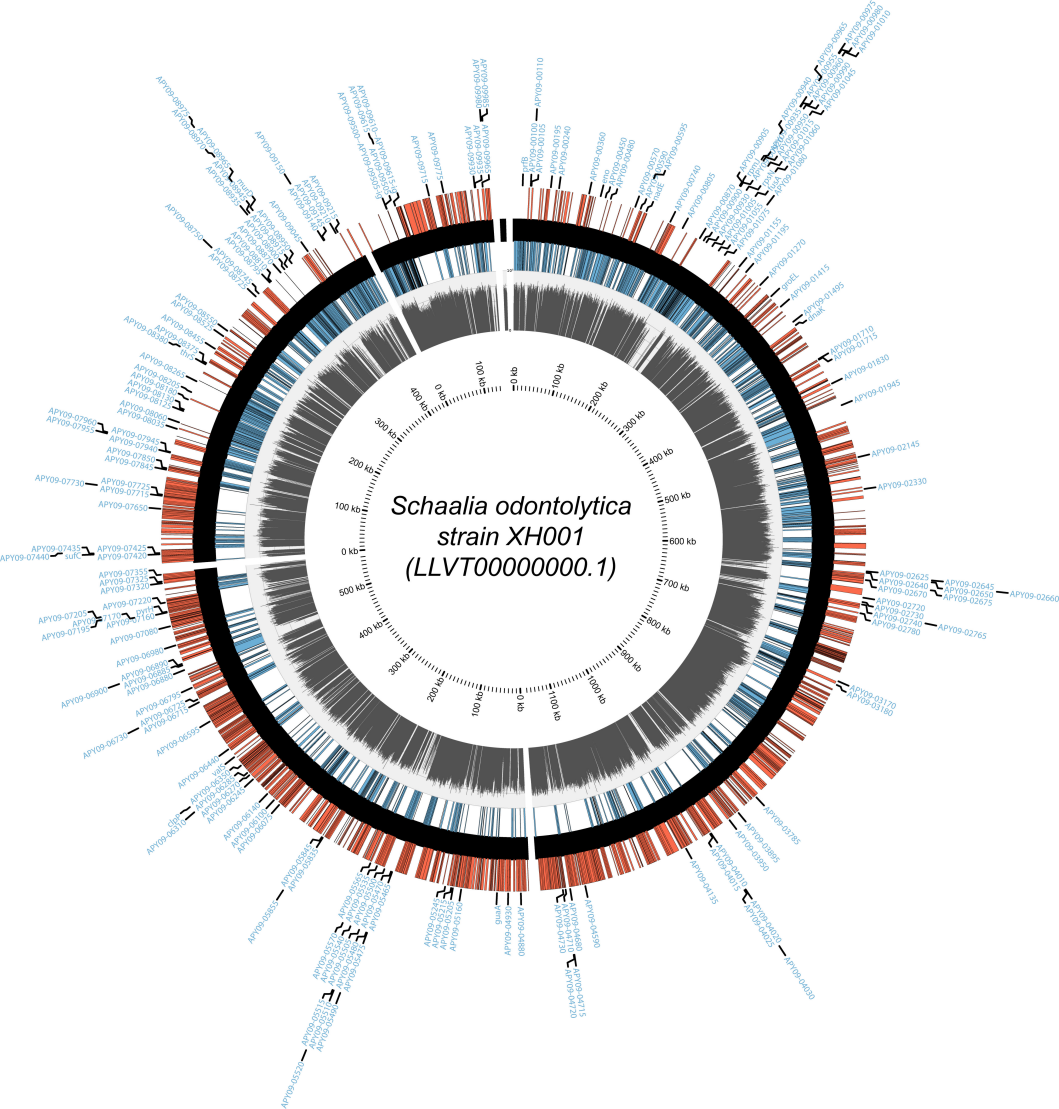
Mapping the putatively essential genes in XH001 for growth on solid media. The total insertion density is log-scaled from 10^0^ to 10^5^ in the gray innermost ring and by strand in the red (+) and dark blue (−) rings. All 203 putatively essential genes are designated by gene name or APY gene loci tags in the light blue outermost ring. The figure was generated using Circos 0.69-9 (https://circos.ca/) (43).

The modified EZ-Tn5 mini-transposon is composed of inverted repeats flanking a constitutive promoter and kanamycin-resistance gene with no transcriptional terminator, thereby creating a polarity effect upon insertion. This polarity often resulted in positional effects of transposon insertions within essential genes and operons. Two examples illustrating these positional effects are shown in Fig. S1. Panel (A) shows that essential gene APY09-00825 (middle gene) tolerates insertions in its 5′ end on the plus strand only, as these result in transcription of the rest of the gene. An alternative Shine-Dalgarno and start codon are located immediately downstream of these insertions. Panel (B) shows that essential operon genes APY09-03775, -03780, and -03785 (middle genes) tolerate insertions in the 3′ end of the first two genes on the plus strand only. This allows for read-through transcription of the remaining genes in the operon. The first two gene products function despite having C-terminal truncations due to the transposon insertions. Based on the polarity of our modified EZ-Tn5 and its associated positional effects, we ignored insertions within the first and last 5% of genes when determining gene essentiality. This stringent selection revealed 203 putatively essential genes (out of 1,936 gene coding sequences) with zero insertions (Fig. 2). Altogether, 10.5% of the XH001 coding genome is predicted to be essential in the BHI growth condition, consistent with previous essential gene studies (31, 54, 56–65).

**Fig 3:**
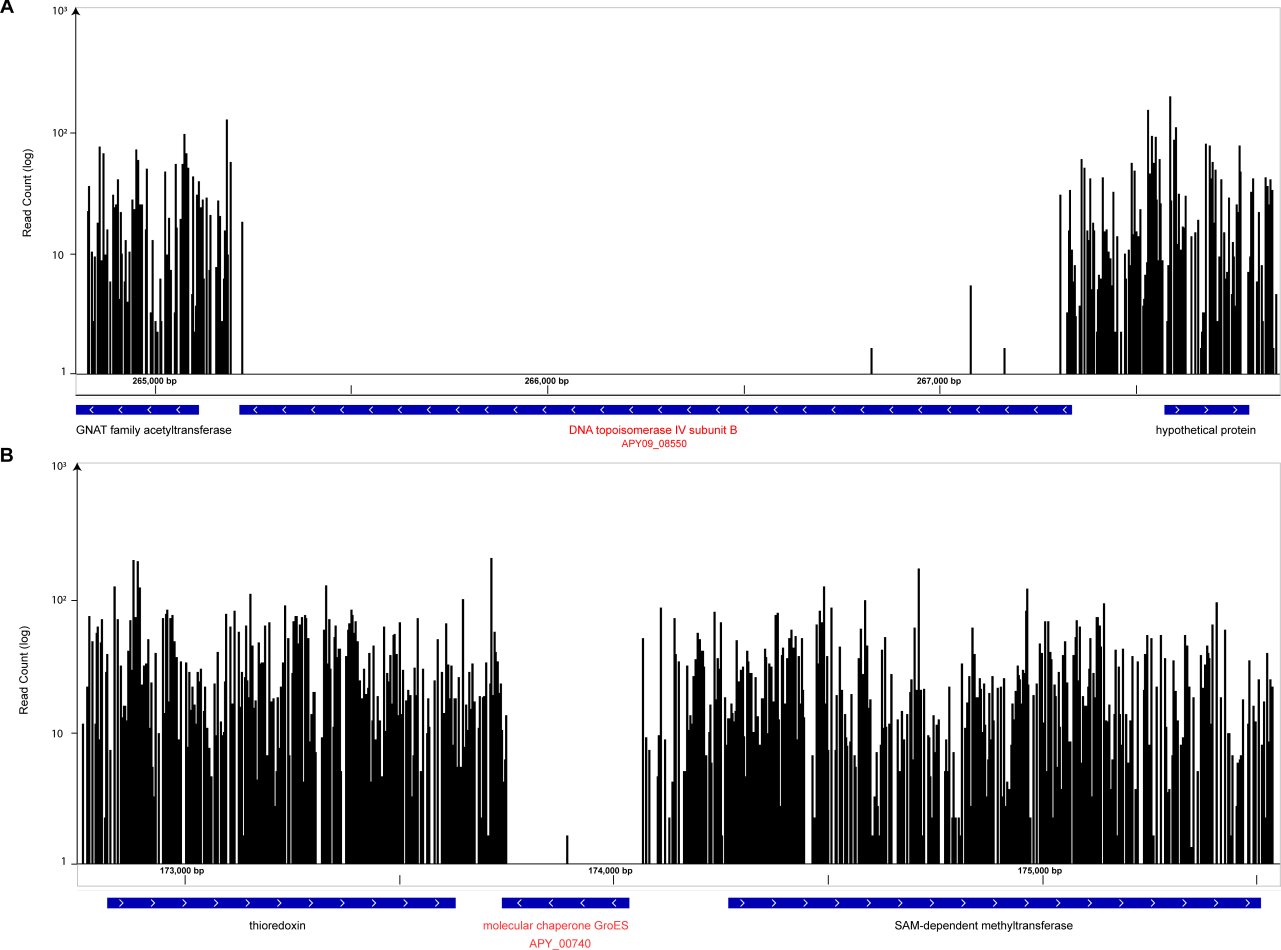
Examples of transposon insertions into highly saturated genes and identification of putatively essential genes. Genome insertion snapshots were taken by displaying unique transposon insertions as a WIG file along the *S. odontolytica* strain XH001 (LLVT01000001.1) genome in Integrated Genome Browser (https://bioviz.org/) (49). Here, genes are displayed as blue arrow boxes in the direction of the coding sequence orientation where black bars represent the genomic position and number of sequenced insertions. (**A**) The genes that encode DNA topoisomerase IV subunit B and (**B**) molecular chaperone GroES have almost zero insertions along their coding sequences, suggesting they are likely to be essential in this growth condition. Moreover, there is a high density of insertions immediately upstream and downstream of these essential genes, signifying non-essential regions.

In a highly saturated transposon library, genes with several unique insertions distributed along the coding sequence are deemed non-essential for the growth condition, whereas genes with no insertions are likely to be essential. Genes that are nearly essential (i.e., that result in a severe growth defect) may have few or no insertions. Moreover, transposon insertions in a gene may exert a polar effect on a downstream essential gene or genes, making it difficult to conclude that the first gene is essential. Therefore, genes that lack insertions within a Tn-seq screen are more correctly referred to as putatively essential. Two examples of genes identified as putatively essential in this study were the genes for chaperone protein DNA topoisomerase IV subunit B (APY09_02670) and GroES (APY09_00740) (Fig. 3). GroES is a chaperonin that is essential in *Escherichia coli* (66). Examination of insertions around *groES* revealed a complete absence of insertions within the coding sequence (Fig. 3A). The degree of sequencing depth and insertion complexity argue against random chance as an explanation for lacking insertions within *groES*. Furthermore, using an aggregated library reduces the probability of observing a lack of insertions due to technical error. Finally, the presence of several unique insertions in the flanking genes rules out the possibility that the region in question is a “cold” spot for transposon insertion, further arguing that the lack of insertions in the coding sequence is due to essentiality. DNA topoisomerase IV subunit B (APY09_02670), which encodes a protein essential for chromosomal segregation, showed a similar pattern (Fig. 3B).

**Fig 4:**
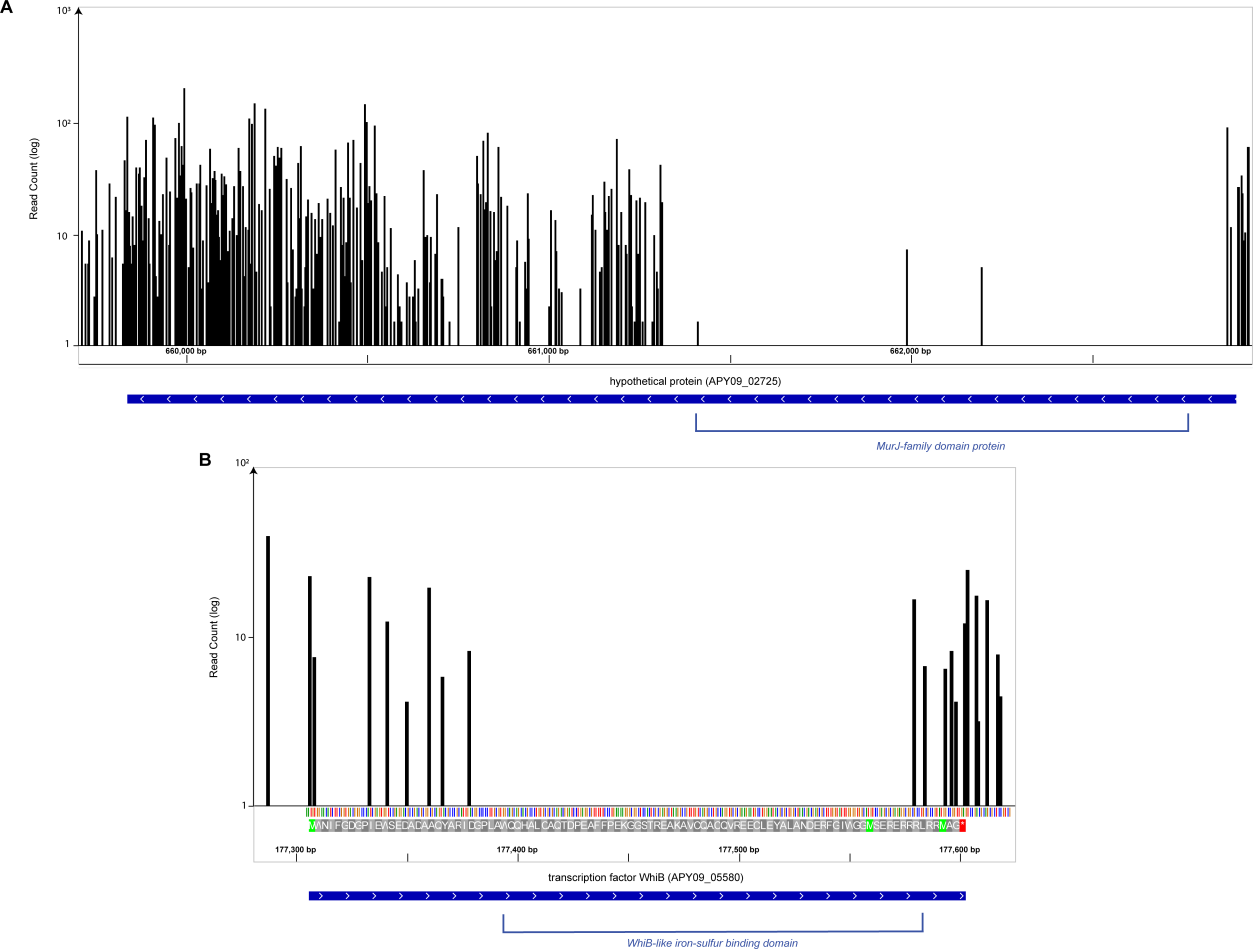
Examples of transposon insertions revealing putatively essential protein domains. The non-uniform distribution of transposon insertions in genes suggests the presence of essential protein domains. Putatively homologous protein functional domains were identified for XH001 proteins using InterProScan (https://www.ebi.ac.uk/interpro/result/InterProScan/#table) (45). (**A**) Locus APY09_02725 encodes a putative protein with several unique insertions, excluding a region predicted to contain a MurJ-family protein domain. (**B**) Transcription factor WhiB (APY09_05580) lacks insertions in its predicted sulfur-binding domain.

In addition to identifying putatively essential genes, a highly saturated transposon insertional library reveals information about additional genetic elements, such as promoter regions, protein functional domains, misannotations, and operon structure. Specific domains within a protein may be essential, even if the overall protein is not, which may be detected by searching for non-uniform distributions of insertions within coding sequences (Fig. 4). For example, examination of insertions within the coding sequence of *whiB* (APY09_05580) revealed a lack of insertions in part of the coding sequence (Fig. 4B). The WhiB protein is a transcription factor that regulates several functions in *Actinobacteria* (67, 68). Examination of the essential protein domain revealed a predicted sulfur-binding domain with the C-X-X-C motif characteristic of WhiB-family proteins (68, 69). Similarly, analysis of coding sequence APY09_02725 revealed a non-uniform distribution in which the first half of the coding sequence lacks any insertion (Fig. 4A). Notably, protein domain prediction software suggests this area is homologous to MurJ-family proteins, which are involved in peptidoglycan synthesis and regulation (70). This may suggest that this locus encodes a protein involved in cell wall regulation, encodes a multi-functional protein, represents a fusion protein between an essential MurJ-family protein and a non-essential protein, or may be a misannotation of the genomes. Altogether, examining insertion distribution within and between predicted coding sequences yields valuable data about genetic structure and protein function.

**Fig 5:**
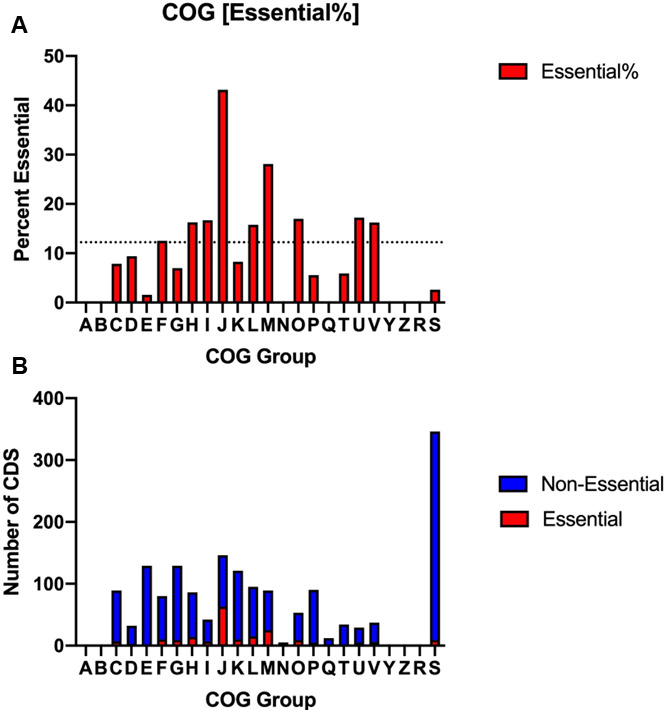
Clusters of orthologous groups in XH001. (**A**) shows the relative distribution of the putatively essential genes in the Clusters of Orthologous Groups (COGs). Each bar represents the relative percent of putatively essential genes within that COG; the dotted line at 12.2% represents the ratio of putatively essential genes with a known COG designation to all coding genes in XH001 with COG designation. (**B**) represents the ratio of putatively essential to non-essential genes with COG designation within each COG group. (**A**) and (**B**) utilize the following *X*-axis key as described: (A = RNA processing and modification; B = chromatin structure and dynamics; C = energy production and conversion; D = cell cycle control, cell division, and chromosome partitioning; E = amino acid metabolism and transport; F = nucleotide transport and metabolism; G = carbohydrate transport and metabolism; H = coenzyme metabolism and metabolism; I = lipid metabolism and metabolism; J = translation ribosomal structure and biogenesis; K = transcription; L = replication, recombination, and repair; M = cell wall/membrane/envelop biogenesis; *N* = cell motility; O = post-translational modification, protein turnover, chaperone functions; *P* = inorganic ion transport and metabolism; Q = secondary metabolites biosynthesis, transport, and catabolism; T = signal transduction mechanisms; U = intracellular trafficking, secretion, and vesicular transport; V = defense mechanisms; W = extracellular structures; Y = nuclear structure; Z = cytoskeleton; R = general functional prediction only; S = function unknown).

The *S. odontolytica* strain XH001 genome used in our analysis has a 1.16 Mb genome and contains 1,998 genes, of which 1,936 are predicted to be protein-coding (10) (GenBank accession: LLVT01000001.1). The functional distribution of the putatively essential genes was determined by assigning genes to COGs as described (42). As expected, a greater percentage of non-essential genes could not be assigned to COGs, resulting in a higher percentage of putatively essential genes when only examining COGs (12.2%) (Fig. 5A; Table S2). Significant enrichment was seen in ‘H’ (coenzyme metabolism and metabolism), ‘I’ (lipid metabolism and metabolism), ‘J’ (translation ribosomal structure and biogenesis), ‘L’ (replication, recombination, and repair), ‘M’ (cell wall/membrane/envelop biogenesis), ‘O’ (post-translational modification, protein turnover, chaperone functions), and ‘U’ (intracellular trafficking, secretion, and vesicular transport). Enrichment in this case was simply defined as a proportion of essential genes in a COG group higher than the ratio of essential COG to nonessential COG. This suggests that the core, which is an essential part of the genome, focuses on these tasks (i.e., lipid metabolism) rather than the other tasks (i.e., amino acid metabolism). Unexpectedly, ‘V’ (defense mechanisms) was also enriched, potentially suggestive of essential stress responses to the microaerophilic conditions during XH001 culturing. Interestingly, ~20% (346) of the total CDS within XH001’s genome are assigned to category ‘S’ (function unknown) (Fig. 5B).

### Comparative analysis of identified putatively essential genes to the validated Database of Essential Genes and predicted essential genes via flux-balance analysis

**Fig 6:**
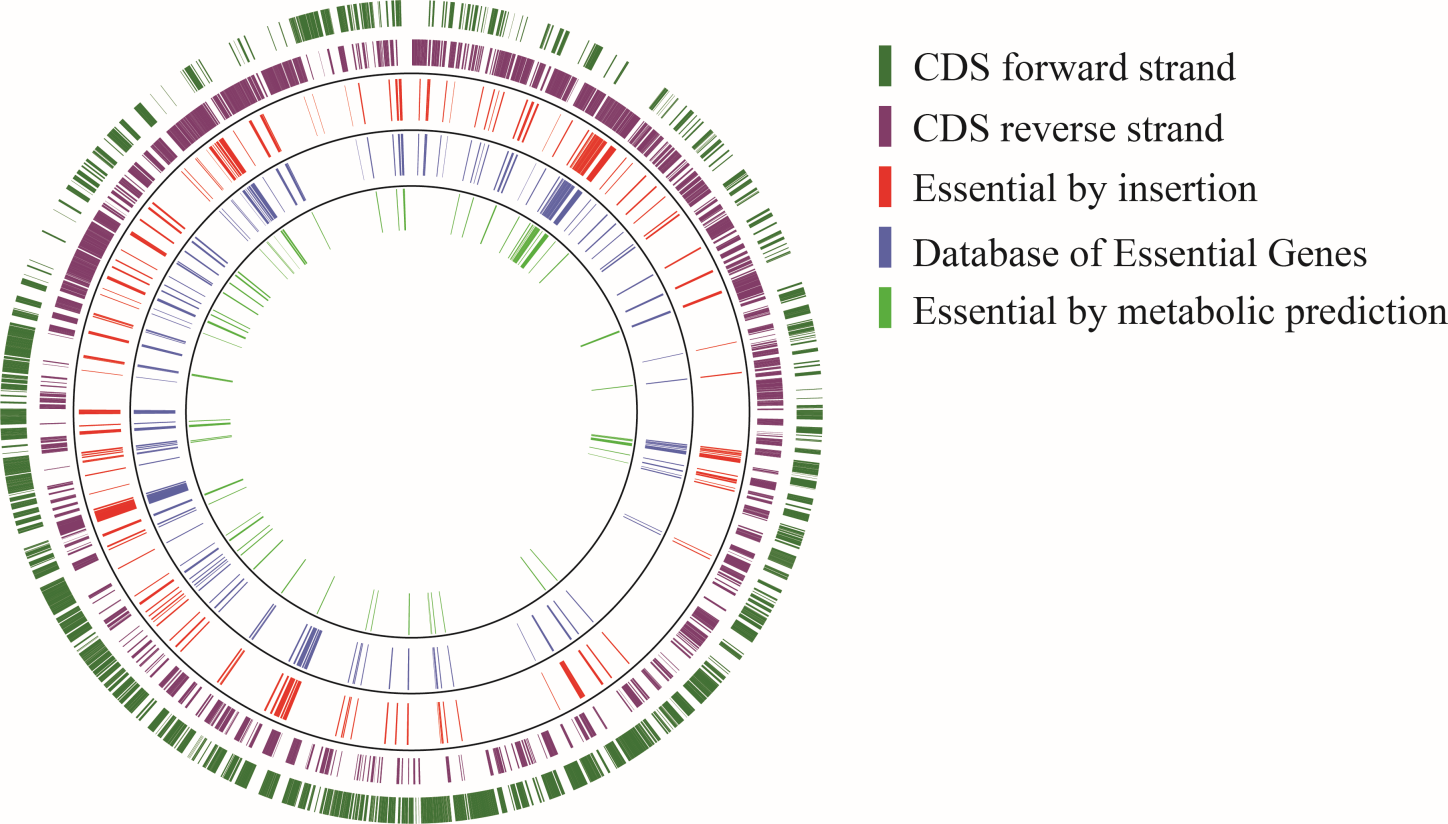
Putatively essential gene comparison to the validated database of essential genes. The figure displays a circular genome plot comparing putatively identified essential genes (CDS) in XH001 (this work) via transposon insertions (red ring) in either the forward (forest green ring) or reverse gene (purple ring) orientation against the experimentally verified Database of Essential Genes (DEG) (blue ring) and juxtaposed against predicted essential genes (using flux-balance analysis) from *S. odontolytica* 17892 within the Bacterial and Viral Bioinformatics Resource Center (sage green ring).

The Dval genome value defined as the number of reads of each gene divided by the expected number of reads based on gene size is the probabilistic essentiality parameter leveraged in this and previous work (31, 35). Three additional analyses were performed to complement this approach and contextualize the putatively essential gene list derived with representative, well-characterized genomes and biologically similar isolates. First, all 203 putatively essential gene coding sequences (CDS) were compared using local BLASTp alignment against proteins in the DEG, a publicly available and updated database of empirically validated essential genes (46). Of the 203 putatively essential CDS in XH001, 193 aligned to known essential genes (Fig. 6). Interestingly, however, 10 predicted proteins have no clear ortholog by BLASTp analysis to DEG proteins (Table S3). We further complemented these results by comparing them against genes computationally predicted to be essential from the Bacterial and Viral Bioinformatics Center (BV-BRC) (71). BV-BRC creates flux-balance models based on ‘reference and representative genomes.’ Genes are then assessed for putative essentiality by examining what their absence does to the flux-balance analysis (71). We then compared the predicted essential genes (300 CDS) for *S. odontolytica* 17982 (a close relative of XH001) through BLASTp analysis against the putatively essential genes of XH001. This analysis revealed an overlap of 93 genes (Table S4) between the DEG analysis and Tn-seq data sets that were both predicted in the BV-BRC and shown to be essential. The 10 orthologs unmatched in the DEG analysis also showed no overlap with the predicted essential genes from the BV-BRC. These putative genes encode proteins that have predicted functionality, including GNAT acetyltransferase activity, iron and redox reaction, and ABC transport, though four putative proteins have no clear function. The final analysis we performed evaluated transcriptomic expression profiles of the 203 putatively essential genes in XH001 during growth in liquid BHI medium by leveraging data sets from two previous studies that investigated global gene expression profiles of XH001 and TM7x during pre-association, the symbiotic (14) and parasitic association phases (13) with TM7x, and the epibiont of XH001 (17) versus XH001 monoculture. This infection model involves MOI-dependent titration of TM7x into XH001 cultures with repeated, serial passaging (*P*) and comparing to XH001 as mono-culture for monitoring (6, 8). The first study sampled during the pre-association and symbiotic phases at P0 or P6 (6 h after culture passage), respectively. The second study evaluated recovery from parasitism by sampling at P4 (at 6, 10, and 15 h after the culture passage). We then evaluated the control XH001 monocultures sampled at those time points for expression of the 203 putatively essential genes. Indeed, all 203 genes were shown to be expressed in all sampled timepoints and growth phases (Table S5).

## DISCUSSION

Essential gene identification studies are important for understanding bacterial physiology and metabolism in natural biological contexts. Here, we present an essential gene study in a *Schaalia* spp. grown on BHI agar (solid media), a rich growth medium, in microaerophilic conditions. We identified 203 putatively essential genes representing 10.5% of the XH001 genes. Unsurprisingly, the putatively essential genes had significant enrichment in basic biological functions, such as lipid metabolism, replication, and ribosomal structure. Ten of these genes have no clear ortholog by BLASTp within the DEG (46) or predicted essential genes in a flux-balance model of the close relative *S. odontolytica* 17982 (Fig. 6). Given the stringency of identifying putatively essential genes in XH001, it is unlikely that these candidates encode non-essential proteins that would not be contained in DEG. We hypothesize that these putatively essential genes may highlight a lack of redundant metabolic pathways or potentially represent unique biologic characteristics of XH001 compared to classic representative species in DEG. Additional genetic and biochemical analyses of these putatively essential genes and proteins would be necessary to confirm their role in XH001. Therefore, we cannot over-interpret these BLASTp results, as most essential genes in the DEG were determined by *in vitro* growth in requisite rich or minimal media (46), not *in situ*. This further highlights major challenges in understanding genetic requirements and biology of microorganisms and is not unique to *Schaalia* spp. since even in the model organism *E. coli*, ~35% of its genome remains to be understood in terms of gene function (72). In this study, as with any using insertional mutagenesis, the possibility exists of having no insertions in a particular gene simply because they are polar on a downstream essential gene, yielding a false positive identification of the gene as putatively essential. Indeed, the modified EZ-Tn5 mini-transposon is composed of inverted repeats flanking a constitutive promoter and kanamycin resistance gene with no transcriptional terminator, thereby creating a polarity effect upon insertion. We confirmed polarity effects of insertions showing toleration in the plus, but not minus, strand in essential genes and operons (Fig. S1). Lastly, we would like to acknowledge that targeted gene knockouts would be the optimal validation of computational predictions and comparative analysis with experimentally validated databases, such as the DEG; however, the scope of this work is to disseminate a new genetic tool, and as a by-product, we identified putatively essential genes for future characterization. Furthermore, attempting mutations in these genes would likely be stymied by generally low gene deletion frequencies, as shown by using current traditional tool capabilities with non-essential genes (7), and probably be unattainable due to the putative requirement for independent growth in BHI. That said, we evaluated the transcriptomic expression profiles of these 203 putatively essential genes in XH001 by referencing published global transcriptomic profiles of XH001 monoculture controls during serial passaging in liquid BHI (13, 14), and we determined that a consistent expression was detected in all 203 genes (Table S5). Future work would not only benefit from individual genetic dissection of these putatively essential genes but also the non-essential genes in XH001, 20% of which also lacked known assigned function (Fig. 5). Leveraging advanced tools, such as CRISPRi (73, 74) or CRISPRi-Tn-seq (75), which currently is lacking for XH001, could be a viable genetic dissection tool where traditional mutagenesis falls short. Together, these data and the XH001 Tn-seq platform presented add a powerful new tool to study the *S. odontolytica* XH001 biology and facilitate a better mechanistic understanding of the epibiotic-parasitic relationship between XH001 and its epibiont, TM7x.

## Data Availability

All unprocessed FASTQ sequencing files are deposited at National Center for Biotechnology Information Sequence Read Archive under BioProject: PRJNA1249110. The Tn-seq analysis code leveraged in this work is deposited here: https://github.com/camillilab/hopcount.

## AFTER EPUB

*[This article was published on 1 August 2025 with incomplete corresponding author designations. The corresponding author designations were updated in the current version, posted on 5 August 2025.]*

## SUPPLEMENTAL MATERIAL

Figure S1 jb.00164-25-s0001.tif Example of putatively essential gene loci illustrating the polarity of the EZ-Tn5 transposon used in this study.

Table S1 jb.00164-25-s0002.xlsx All bacterial strains, plasmids, and media used in this work.

Table S2 jb.00164-25-s0003.xlsx 203 putatively essential genes discovered in this work.

Table S3 jb.00164-25-s0004.xlsx 10 putatively essential genes with no BLASTp homolog nor in DEG.

Table S4 jb.00164-25-s0005.xlsx 93 overlapping putatively essential genes between DEG and BV-BRC analyses, and Tn-seq datasets in this work.

Table S5 jb.00164-25-s0006.xlsx Transcriptomic datasets (Hendrickson et al., 2022; Hendrickson et al., 2024) of all putatively essential genes discovered in this work.

ASM does not own the copyrights to Supplemental Material that may be linked to, or accessed through, an article. The authors have granted ASM a non-exclusive, world-wide license to publish the Supplemental Material files. Please contact the corresponding author directly for reuse.
