# Supplementary figures and images for: Identifying essential genes in Schaalia odontolytica using a saturated transposon library
Source: J Bacteriol. 2025 Aug 1;207(8):e00164-25. doi: 10.1128/jb.00164-25 (PMC12369343; doi:10.1128/jb.00164-25)

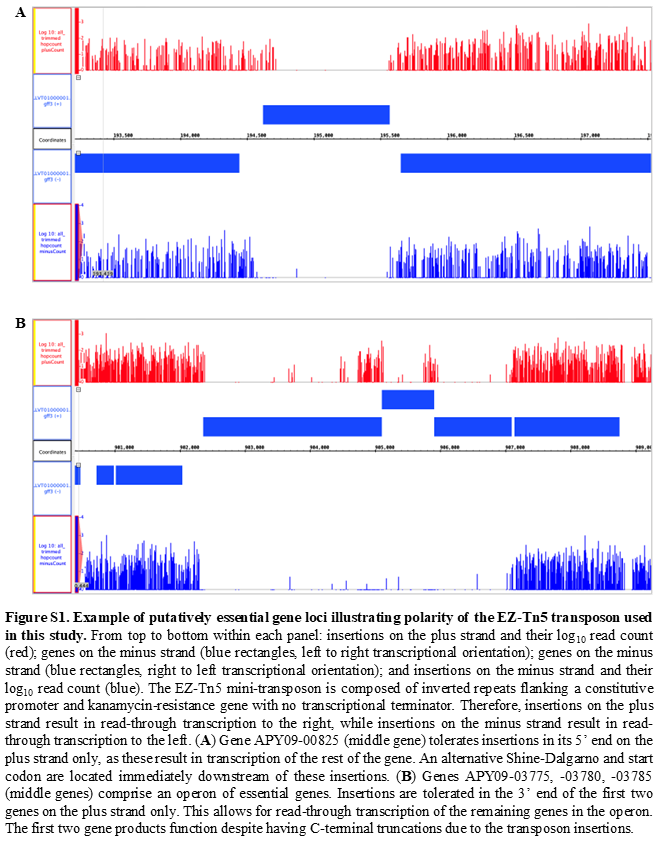

Supplement: Figure S1 — Example of putatively essential gene loci illustrating the polarity of the EZ-Tn5 transposon used in this study. [file jb.00164-25-s0001.tif]
